# Treatment outcome of high-dose image-guided intensity-modulated radiotherapy using intra-prostate fiducial markers for localized prostate cancer at a single institute in Japan

**DOI:** 10.1186/1748-717X-7-105

**Published:** 2012-07-06

**Authors:** Ken Takeda, Yoshihiro Takai, Kakutaro Narazaki, Masatoshi Mitsuya, Rei Umezawa, Noriyuki Kadoya, Yukio Fujita, Toshiyuki Sugawara, Masaki Kubozono, Eiji Shimizu, Keiko Abe, Yuko Shirata, Yohjiro Ishikawa, Takaya Yamamoto, Maiko Kozumi, Suguru Dobashi, Haruo Matsushita, Koichi Chida, Shigeto Ishidoya, Yoichi Arai, Keiichi Jingu, Shogo Yamada

**Affiliations:** 1 Department of Radiological Technology, School of Health Sciences, Faculty of medicine, Tohoku University, Sendai, Japan; 2 Department of Radiology and Radiation Oncology, Hirosaki University School of medicine, Hirosaki, Japan; 3 Department of Radiation Oncology, Sendai medical Center, Sendai, Japan; 4 Sendai Radiation Oncology and Imaging Clinic, Sendai, Japan; 5 Department of Radiation Oncology, Tohoku University Hospital, Sendai, Japan; 6 Department of Urology, Tohoku University Hospital, Sendai, Japan

**Keywords:** Image-guided radiotherapy, Prostate cancer, Biochemical control, Toxicity

## Abstract

**Background:**

Several studies have confirmed the advantages of delivering high doses of external beam radiotherapy to achieve optimal tumor-control outcomes in patients with localized prostate cancer. We evaluated the medium-term treatment outcome after high-dose, image-guided intensity-modulated radiotherapy (IMRT) using intra-prostate fiducial markers for clinically localized prostate cancer.

**Methods:**

In total, 141 patients with localized prostate cancer treated with image-guided IMRT (76 Gy in 13 patients and 80 Gy in 128 patients) between 2003 and 2008 were enrolled in this study. The patients were classified according to the National Comprehensive Cancer Network-defined risk groups. Thirty-six intermediate-risk patients and 105 high-risk patients were included. Androgen-deprivation therapy was performed in 124 patients (88%) for a median of 11 months (range: 2–88 months). Prostate-specific antigen (PSA) relapse was defined according to the Phoenix-definition (i.e., an absolute nadir plus 2 ng/ml dated at the call). The 5-year actuarial PSA relapse-free survival, the 5-year distant metastasis-free survival, the 5-year cause-specific survival (CSS), the 5-year overall survival (OS) outcomes and the acute and late toxicities were analyzed. The toxicity data were scored according to the Common Terminology Criteria for Adverse Events, version 4.0. The median follow-up was 60 months.

**Results:**

The 5-year PSA relapse-free survival rates were 100% for the intermediate-risk patients and 82.2% for the high-risk patients; the 5-year actuarial distant metastasis-free survival rates were 100% and 95% for the intermediate- and high-risk patients, respectively; the 5-year CSS rates were 100% for both patient subsets; and the 5-year OS rates were 100% and 91.7% for the intermediate- and high-risk patients, respectively. The Gleason score (<8 vs. ≥8) was significant for the 5-year PSA relapse-free survival on multivariate analysis (p = 0.044). There was no grade 3 or 4 acute toxicity. The incidence of grade 2 acute gastrointestinal (GI) and genitourinary (GU) toxicities were 1.4% and 8.5%, respectively. The 5-year actuarial likelihood of late grade 2–3 GI and GU toxicities were 6% and 6.3%, respectively. No grade 4 GI or GU late toxicity was observed.

**Conclusions:**

These medium-term results demonstrate a good tolerance of high-dose image-guided IMRT. However, further follow-up is needed to confirm the long-term treatment outcomes.

## Background

Recently, several studies have confirmed the advantages of delivering high doses of external beam radiotherapy (EBRT) to achieve optimal tumor-control outcomes in patients with localized prostate cancer. It is now clear that conventional EBRT doses in the range of 70 Gy are not sufficient to eradicate local prostate disease [1,2]. In addition, a higher EBRT dose requires greater accuracy and precision. Thus, various position verification methods, including image-guided radiotherapy, have been developed, and their effectiveness has been reported [3]. However, there are few publications regarding the treatment outcome after completion of the delivery of high-doses with image-guided intensity-modulated radiotherapy (IMRT) using daily intra-prostatic fiducial marker-based position verification for localized prostate cancer [4].

We investigated the medium-term treatment outcomes, including the 5-year prostate-specific antigen (PSA) relapse-free survival rate, the 5-year distant metastasis-free survival rate, the 5-year cause-specific survival (CSS) rate, the 5-year overall survival (OS) rate and the toxicity outcomes, after high-dose image-guided IMRT using daily intra-prostatic fiducial markers in patients with clinical localized prostate cancer.

## Methods

Between 2003 and 2008, 150 patients with localized prostate cancer were treated with image- guided IMRT at Tohoku University Hospital. Among these patients, 141 patients who satisfied the eligibility criteria were included in the current study and retrospectively analyzed.

### Eligibility criteria

The eligible patients had a biopsy-confirmed adenocarcinoma of the prostate with the clinical stage T1-3N0M0 and were classified in the National Comprehensive Cancer Network (NCCN)-defined ( http://www.nccn.com) intermediate- or high-risk groups. Each patient received magnetic resonance imaging before the initial treatment to exclude lymph node metastasis and for the staging procedure. Patients with T2b or T2c clinical stage tumors, a Gleason score (GS) of 7, or a pretreatment PSA level between 10 and 20 ng/mL were classified as intermediate-risk disease. Patients who had tumors with a T3a clinical stage or higher, a GS ≥8, or a pretreatment PSA level >20 ng/mL were classified as having high-risk disease.

### Exclusion criteria

NCCN-defined low-risk patients with a T1-2a clinical stage tumor, a GS <7, a pretreatment PSA level <10 ng/mL, and N1 disease were not enrolled in this study. Patients with a T4 clinical stage tumor, the presence of metastasis, other concurrent invasive cancers, or active collagen disease were also not included. Additionally, patients with salvage intent were not enrolled, including patients with a biochemical relapse following a prior prostatectomy, prior pelvic radiotherapy, and hormonal therapy. Patients with a follow-up period within 1 year were also not registered in this analysis.

A total of 105 patients with high-risk localized prostate cancer and 36 patients with intermediate-risk localized prostate cancer received IMRT up to a prescribed dose of 76 Gy or 80 Gy and were investigated. The patient characteristics are listed in Table 1. Pelvic lymph node dissection (PLND) was performed in 45 patients (32%) to rule out metastatic disease.

**Table 1:** Patient characteristics

**Age (y)**			**Median (range)**	**71 (50–83)**
			**N**	**%**
T stage	T1		34	24
	T2		40	28
	T3		67	48
Gleason score	<8		73	52
	8 − 10		68	48
Initial PSA	≤20		93	66
	>20		48	34
NCCN riskGroup	IR		36	26
	HR		105	74
ADT		Yes	124	88
		IR	24	17
		HR	100	71
		STADT	27	22
		LTADT	97	78
		No	17	12
Diabetes			23	16
Hypertension			50	35
Hemorrhoid			37	26

*Abbreviations*: PSA, prostate-specific antigen; NCCN, national comprehensive cancer network; IR, intermediate risk; HR, high risk; ADT, androgen-deprivation therapy; STADT, short-term ADT; LTADT, long-term ADT.

All eligible patients provided written informed consent before treatment. The institutional research ethics board approved this study.

### Radiotherapy

The detailed techniques for IMRT treatment planning and delivery have been previously reported [5]. Briefly, each patient was implanted with three gold fiducial markers in the prostate gland before the treatment-planning computerized tomography (CT) scan was acquired. All patients were immobilized in the supine position with a vacuum bag system for their entire body. Since January 2004, we have temporarily used a urethral catheter for identification of the urethra on radiotherapy-planning CT images and for contouring the urethra after image acquisition. The CT scans were then performed at a 2.5-mm slice thickness. In the IMRT planning, Eclipse (release 6.5; Varian medical Systems, Palo Alto, CA, USA) was used for dose calculations. The clinical target volume (CTV) included the prostate and seminal vesicles. To adequately encompass the extent of tumor invasion in the seminal vesicles, the CTV involved the base of the seminal vesicles in T1-3a patients and more distal to entire portion of the seminal vesicles in T3b patients, respectively. Based on our previous study [6], the CTV was expanded in three dimensions with a 0.5-cm margin to obtain the planning target volume (PTV) with the exception of the prostate–rectum interface, where a 0.3-cm margin was adopted to decrease rectal involvement. A portion of the rectal wall located at the level of the PTV and 0.5 cm outside of the PTV on the CT images was contoured. The rectum, bladder, bowel, and femur were contoured as critical normal tissue structures. The rectal wall was defined with a 2-mm internal wall extraction. The bladder was entirely contoured, and a 5-mm inner wall defined the bladder wall volume.

IMRT was delivered using 15MV photons generated by a Clinac 23 EX linear accelerator (Varian medical Systems, Palo Alto, CA). The standard 5–8 coplanar beams were used. The prescribed dose used to cover 95% of the target volume (D_95_) was 76 Gy in 13 patients and 80 Gy in 128 patients. A total dose of 76 Gy in 2-Gy daily fractions was delivered to 13 patients who had other prior severe diseases, including diabetes or cardiovascular disorders. The maximum dose heterogeneity allowable in the PTV was 10%. Each treatment plan was optimized to ensure the following conditions: no more than 65% of the rectal and urinary bladder wall received >35 Gy (V_35_ ≤ 65%); no more than 45% of the rectal and urinary bladder wall received >55 Gy (V_55_ ≤ 45%); no more than 25% of the rectal and urinary bladder wall received >75 Gy (V_75_ ≤ 25%); and the urethral, rectal, and bladder walls received no more than 80 Gy. In the overlap region between the PTV and these critical organs, the constraint was set to 95% of the prescription dose for the rectum and 95% for the urethra. The latter dose constraint for the urethra has been applied to 108 patients since January 2004.

In addition, prior to the acquisition of the treatment planning CT images and 30 minutes before the daily IMRT, each patient urinated to ensure the bladder was in the same state. In addition, the patients emptied their bowels just before the daily IMRT. For every treatment fraction, the patients were initially prepared using laser marks on their skin, and they were then repositioned using the Varian On-Board Imager based on the positions of the three intra-prostatic fiducial markers, and the precision-of-position verification was within 1 mm. We did not find any instances of fiducial migration during treatment. All patients were treated to their prescribed dose in daily 2.0 Gy fractions. The imaging dose was not accounted for in the treatment plans.

### Hormonal therapy

Androgen-deprivation therapy (ADT) was used at the discretion of the treating physician. ADT primarily consisted of an oral anti-androgen and luteinizing hormone-releasing hormone agonist administered as depot injections. In 124 patients receiving ADT, the median ADT duration was 11 months (range: 2–88 months). Among these patients, 44 (35%) received only neoadjuvant ADT, and the other 80 (65%) were treated with neoadjuvant ADT, concurrent and adjuvant ADT. Table 1 shows the details of the ADT duration. ADT was classified as short-term (STADT) if it was administered for ≤6 months and long-term (LTADT) if it was administered for >6 months. The median duration of ADT in the 24 intermediate-risk and 100 high-risk patients was 5 months (range: 4–32 months) and 12 months (range: 2–88 months), respectively. The details of STADT and LTADT are as follows: for the 24 intermediate-risk patients, there were 10 STADT and 14 LTADT, and for the 100 high-risk patients, there were 17 STADT and 83 LTADT. The median duration between the initiation of ADT and the start of IMRT was 8 months (range: 1–36 months).

### After treatment follow-up evaluation

Follow-up evaluations after the completion of treatment were performed at 3- to 6-month intervals for 5 years and every 6 months thereafter. The median follow-up was 66 months (range: 17–111 months).

Freedom from biochemical relapse was analyzed using the Phoenix consensus definition (i.e., an absolute nadir PSA level plus 2 ng/mL more than the recorded level) [7,8]. For CSS analysis, patients with documentation of biochemical or metastatic relapsed disease who subsequently died were scored as deaths from localized prostate cancer.

Acute and late toxicity data were scored according to the National Cancer Institute-designated Common Terminology Criteria for Adverse Events Version 4.0.

### Statistical analyses

The distributions of the 5-year PSA relapse-free survival were calculated using Kaplan–Meier curves for biochemical control using the one failure definition. The 5-year actuarial distant metastasis-free survival, CSS, and OS rates were also evaluated by Kaplan–Meier curves. Univariate analyses (UA) and multivariate analyses (MA) were performed to determine the related PSA relapse-free survival predictors (i.e., NCCN risk stratification, GS, ADT use and duration, STADT, LTADT, pretreatment PSA, and PLND) and the late gastrointestinal (GI) and genitourinary (GU) side effects ≥ grade 2 (i.e., age, ADT use, duration between ADT initiation and the start of IMRT, the presence of diabetes, hypertension, hemorrhoids, acute grade 2 GI and GU toxicities, and prescribed RT dose). MA was performed using a Cox regression model. Statistical analyses were performed using the Statistical Package for Social Sciences for Windows, version 20. A *p*-value <0.05 (two-sided) was considered to be statistically significant in all tests.

## Results

### Biochemical tumor-control rate

**Figure 1:**
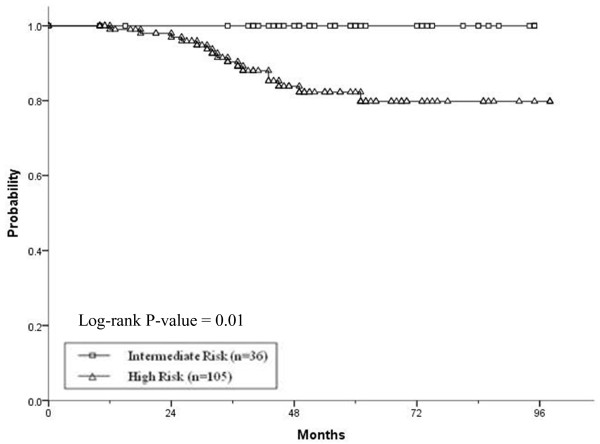
**Phoenix consensus definition PSA relapse-free survival by NCCN risk group. ***Abbreviations*: PSA, prostate-specific antigen; NCCN, national comprehensive cancer network.

The 5-year actuarial PSA relapse-free survival outcomes for the intermediate- and high-risk groups were 100 and 82.2%, respectively [Figure 1], and the NCCN risk classification, GS, and pretreatment PSA were significant in UA, whereas only the GS was statistically significant variable in MA [Table 2]. 

**Table 2:** **Statistical analyses of predictors for the 5-year PSA relapse-free survival, *p *values**

	**UA**	**MA**
NCCN risk (IR vs. HR)	0.01^*^	0.96
GS (8–10 vs. < 8)	<0.001^*^	0.044^*^
ADT (yes vs. no)	0.1	0.967
ADT duration (continuous)	0.452	0.549
STADT vs. LTADT	0.477	0.513
Pretreatment PSA (≤20 vs. >20)	0.046^*^	0.29
PLND (yes vs. no)	0.316	0.454

*Abbreviations*: UA, univariate analysis; MA, multivariate analysis; ^*^, statistical significance; PLND, pelvic lymph node dissection; other abbreviations as in Table 1.

### Distant metastasis-free survival, cause-specific survival and overall survival rates

**Figure 2:**
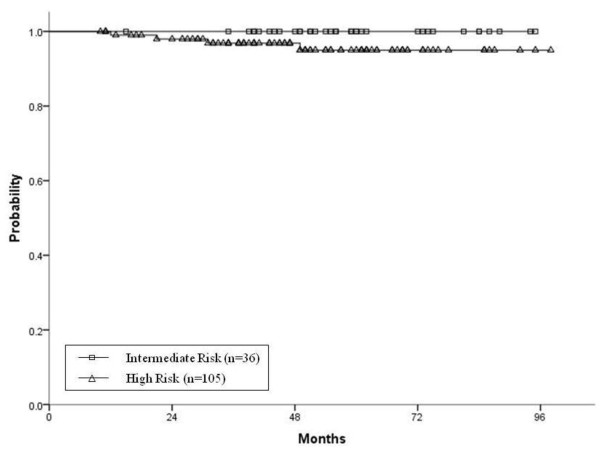
The Kaplan-Meier curve illustrates the actuarial probability of achieving distant metastasis-free survival.

Distant metastases developed in four (2.8%) patients. The 5-year actuarial distant metastasis-free survival rates for the intermediate- and high-risk groups were 100 and 95%, respectively [Figure 2]. The 5-year CSS rates for the intermediate- and high-risk patients were both 100%. The 5-year OS rates for the intermediate- and high-risk patients were 100 and 91.7%, respectively.

### Toxicity

Grades 1 and 2 GI acute toxicity were identified in 29 (20%) and 2 (1.4%) patients, respectively. Grades 1 and 2 acute GU toxicities occurred in 84 (60%) and 12 (8.5%) patients, respectively. There was no acute GI and GU toxicity grade 3 or higher.

**Figure 3:**
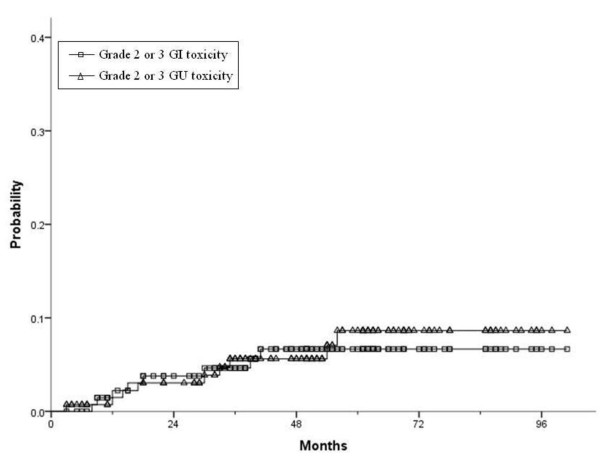
**Kaplan-Meier actuarial probability of late grade 2 or 3 GI and GU toxicities. ***Abbreviations*: GI, Gastrointestinal; GU, Genitourinary.

Late grade 2 or 3 GI toxicities developed in eight patients (5.7%) at a median of 18 months after image-guided IMRT. There was rectal hemorrhage in seven patients and proctitis in one patient. Rectal hemorrhage as a grade 3 late GI toxicity occurred in two patients who were treated with several transfusions and a laser cauterization procedure. No grade 4 or greater GI complications were observed. The 5-year actuarial likelihood of late grade 2 or 3 GI toxicities was 6% [Figure 3]. In UA, the group without ADT was related to grade 2 or 3 late GI toxicities, whereas that relationship was not significant in MA [Table 3]. In addition, the duration between ADT initiation and the start of IMRT was also not statistically significant for late GI toxicities grade 2 or 3 [Table 3]. 

**Table 3:** **Statistical analyses of predictors for late toxicity, *p *values**

	**Late grade 2 or 3 GI toxicity**		**Late grade 2 or 3 GU toxicity**	
	**UA**	**MA**	**UA**	**MA**
Age (>71)	0.414	0.42	0.078	0.039^*^
ADT	0.025^*^	0.401	0.889	0.314
Duration between ADT initiation and the start of IMRT	0.088	0.619	0.522	0.312
Diabetes	0.526	0.877	0.66	0.511
Hypertension	0.396	0.474	0.38	0.732
Hemorrhoid	0.952	0.853	NA	NA
Acute grade 2 GI toxicity	0.743	0.992	NA	NA
Acute grade 2 GU toxicity	NA	NA	<0.001^*^	0.001^*^
76 Gy vs. 80 Gy	0.77	0.716	0.291	0.987

*Abbreviations*: *G*I, gastrointestinal; GU, genitourinary; NA, not applicable; other abbreviations as in Tables 1– 2.

Late grade 2 or 3 GU toxicities developed in nine patients (6.4%) at a median of 30 months after image-guided IMRT. There was urinary retention in four patients, urinary frequency in two patients, urinary tract obstruction in one patient, urgency in one patient and non-infective cystitis in one patient. No grade 4 or greater GU toxicity was observed. The 5-year actuarial likelihood of late grade 2 or 3 GU toxicities was 6.3% [Figure 3]. In UA and MA, acute grade 2 GU toxicity was predictive of late grade 2 or 3 GU toxicities [Table 3]. Although age (>71) was not significant in UA, it was predictive in the MA [Table 3].

## Discussion

Our results show that high-dose image-guided IMRT can be safely performed and is well tolerated. This study also demonstrated relatively few biochemical failures after image-guided IMRT in patients with intermediate- and high-risk localized prostate cancer as a medium-term period assessment [Table 4]. Nevertheless, it is difficult to tell whether this was a function of high-dose image-guided IMRT, heterogeneity within this series of patients, different hormonal therapies, or any combination of these factors.

**Table 4:** Literature review

	**RT dose (Gy)**		**ADT rate (%)**	**ADT duration (M)**	**PSA relapse-free survival (%)**	
Alicikus et al. [1]	81	LR	54 (total)	3	PD: 10-year	81
		IR				78
		HR				62
Martin et al. [4]	79.8	LR	13.6	not mentioned	PD: 5-year	88.4
		IR	11.0			76.5
		HR	45.9			77.9
Zelefsky et al. [12]	81	LR	33.5	3	AD: 8-year	85
		IR	52			76
		HR	92			72
		LR	33.5		PD: 8-year	89
		IR	52			78
		HR	92			67
Cahlon, et al. [16]	86.4	LR	66 (total)	3 or 9	PD: 5-year	98
		IR				85
		HR				70
Current study	80 or 76	IR	67	5 (median) (range: 4–32)	PD: 5-year	100
		HR	95	12 (median) (range: 2–88)		82.2

*Abbreviations*: RT, radiotherapy; M, months; LR, low risk; PD, Phoenix Consensus definition; AD, American Society for Therapeutic Radiology and Oncology definition; other abbreviations as in Table 1.

There is clear support for dose escalation in prostate cancer radiotherapy. Several studies have confirmed that 74- to 81-Gy doses provided a 15-20% improvement in biochemical control compared with conventional doses of <70 Gy [9-12]. According to previous studies, biochemical tumor-control rate improvement will lead to better distant metastasis-free survival and CSS [13-15]. The current distant metastasis-free survival and CSS rates were at least equivalent to other reports, although direct comparison with other publications is difficult due to differences in the follow-up periods [1,2,12].

In addition to high-dose image-guided IMRT, a relatively high rate and long duration of ADT administration may contribute to the current biochemical tumor-control outcome, although the effect was not statistically significant [Tables 2 and 4[1,12,16,17]. To our knowledge, few publications have reported high-dose EBRT with a high rate and long ADT administration duration that was similar to this study. Moreover, according to D’Amico et al. and Bolla et al., the radiotherapy survival benefit may be improved by adding ADT for high- and intermediate-risk localized prostate cancer [17,18]. Furthermore, a meta-analysis of Radiation Therapy Oncology Group prostate cancer trials demonstrated that STADT appeared to improve the 8-year disease-specific survival for intermediate-risk patients, whereas LTADT improved the 8-year overall survival for high-risk patients [19]. According to Alicikus et al., the lack of a benefit from ADT in their high-risk patients may have been caused by the relatively short course of only 5–6 months of ADT in their study [1]. They advocated the use of a longer ADT course, particularly for high-risk patients.

The incidence of late grade ≥2 GI and GU toxicity following high-dose radiotherapy in recent studies ranged from 3.7 to 22% and from 8.6 to 35%, respectively [1,4,16,20]. In this report, the likelihood of developing late grade ≥2 GI and GU toxicities was 6 and 6.3%, respectively, at 5 years. One reason that may explain our late GI toxicity incidence may be that we did not confirm the actual position of the rectum during the entire IMRT period, although every patient was prepared before each treatment with minimized bowel contents, including gas and stool, which was similar to the acquisition of treatment-planning CT. Although Smeenk et al. reported some procedures for reducing the physiological bowel effects, those techniques were not used in this study [21].

The relationship between ADT administration and late grade 2–3 GI toxicities was not statistically significant in MA, whereas there appeared to be a relationship in UA [Table 3. In addition, the duration between ADT initiation and the start of IMRT was not correlated with late GI grade 2 or 3 toxicities [Table 3. Although there may be some mechanisms that explain why patients with ADT administration demonstrated a low tendency for late grade 2–3 GI toxicities in UA, it is difficult to clarify. This topic is controversial. Several authors reported that ADT administration increases late GI toxicity [20,22]. In contrast, other studies have indicated lower GI and GU toxicity rates when ADT was added to EBRT for localized prostate cancer [23,24]. Longer follow-up and further investigation are needed.

The incidence of late grade 2–3 GU toxicities appeared to be lower than that previously reported [1,4,16,20]. Several reasons may exist. One reason might be that we set the urethral dose constraints to reduce the maximal urethral dose to <80 Gy in patients treated with 80 Gy since January 2004. Zelefsky et al. indicated more severe late urinary toxicity following high-dose IMRT delivering 81.0 Gy or 86.4 Gy [25]. According to these authors, the 3-year actuarial likelihood of late grade ≥2 urinary toxicities was 15%. Therefore, we began setting the urethral dose constraints in 2004. Another reason might be that our image-guided IMRT had a precision-of-position verification within 1 mm. However, further follow-up and examination is necessary to evaluate late GU toxicity and related factors.

In this series, acute grade 2 GU toxicity was statistically related to late grade 2–3 GU toxicity in the MA [Table 3. Alicikus et al. also demonstrated that acute grade ≥2 GU toxicity was predictive for late grade ≥2 GU toxicity [1]. According to Bolla et al. and Peeters *et al*., an increase in late GU toxicity was associated with LTADT in addition to RT [17,22]. In contrast, similar to Alicikus et al., the use and duration of ADT was not related to the late grade 2–3 GU toxicity in this study [1] [Table 3.

Grade 2 acute GI and GU symptoms developed in two (1.4%) and 12 patients (8.5%), respectively. There was no acute GI or GU toxicity of grade 3 or higher. Similar to Alicikus et al., the acute GI toxicity was minimal [1]. However, the current incidence of acute GU symptoms was higher than that in their study [1]. Alicikus et al. reported a grade 2 acute GU toxicity frequency of 3%. Nevertheless, other studies have indicated higher occurrences of grade 2 acute GU toxicity than our study, ranging from 22 to 47% [3,4,16]. Accordingly, our results, with respect to acute and late GI and GU toxicities, appear to be favorable.

Several limitations exist in this study. First, this study had no randomization; therefore, it had an inherent potential for selection bias. Additionally, our sample size was more limited than some other studies [1,4,12,16]. Second, this analysis included a small number of patients who were followed for <5 years, which did not allow an adequate period to evaluate clinical failures or side effects. The small number of biochemical failures could be a function of the short follow-up. Third, we unfortunately did not estimate sexual function before and after treatment in this analysis, whereas several authors have reported a relationship between ADT use and the development of erectile dysfunction [1,26,27]. However, we reported the health-related quality of life in smaller numbers of patients treated with image-guided IMRT [28], and further investigation is in progress.

## Conclusions

We report the medium-term treatment outcomes of high-dose image-guided IMRT for patients with localized prostate cancer. The current treatment is well tolerated and appears to provide valuable biochemical tumor control. Nevertheless, a relatively high rate and long duration of ADT administration may have also potentially contributed to the outcome. Further investigation is needed to optimize integration between dose-escalated radiotherapy and adequate ADT. Additionally, longer follow-up is essential to evaluate the long-term treatment outcomes.

## Abbreviations

EBRT: External Beam Radiation Therapy; IMRT: Intensity-Modulated Radiation Therapy; PSA: Prostate-Specific Antigen; CSS: Cause-Specific Survival; OS: Overall Survival; NCCN: National Comprehensive Cancer Network; GS: Gleason Score; PLND: Pelvic Lymph Node Dissection; CT: Computerized Tomography; CTV: Clinical Target Volume; PTV: Planning Target Volume; ADT: Androgen-Deprivation Therapy; STADT: Short-Term ADT; LTADT: Long-Term ADT; UA: Univariate Analysis; MA: Multivariate Analysis; GI: Gastrointestinal; GU: Genitourinary.

## Competing interests

The authors declare that they have no competing interests.

## Authors contributions

Study design: YT, KN, MM, SI, YA. Patient contribution, manuscript review: KT, YT, KN, MM, RU, NK, YF, TS, MK, ES, KA, YS, YI, TY, MK, SD, HM, KC, SI, YA, KJ, SY. Data collection: KT, YT, KN, RU, TS, MK, ES, KA, YS, YI, TY, MK, HM, SI, YA. Data analysis, manuscript preparation: KT. All authors reviewed and approved the final manuscript.
